# Supplementary material for: Association of low blood pressure and falls: An analysis of data from the Leiden 85-plus Study
Source: PLoS One. 2023 Dec 20;18(12):e0295976. doi: 10.1371/journal.pone.0295976 (PMC10732458; doi:10.1371/journal.pone.0295976)
Supplement: S1 File — (DOCX) [file pone.0295976.s001.docx]

**Association of low blood pressure and falls:**

**An analysis of data from the Leiden 85-plus Study**

***Supporting material***

David Röthlisberger^1¶^, Katharina Tabea Jungo^1¶^, Lukas Bütikofer^2^, Rosalinde K E Poortvliet^3^, Jacobijn Gussekloo^3,4^, Sven Streit^1*^

^1^ Institute of Primary Health Care (BIHAM), University of Bern, Bern, Switzerland

^2^ CTU Bern, University of Bern, Bern, Switzerland

^3^ Department of Public Health and Primary Care, Leiden University Medical Center, Leiden, the Netherlands

^4^ Department of Internal Medicine, Section Gerontology and Geriatrics, Leiden University Medical Center, Leiden, the Netherlands

^¶^ These authors contributed equally to this work

**Table of content**

[S1 Fig. Plot showing the association between blood pressure and falls - Spline models. 2](#_Toc152938197)

[S2 Fig. Plot showing the association between blood pressure and the probability of falls, adjusted by year 3](#_Toc152938198)

[S3 Fig. Plot showing the association between the use of antihypertensive medications and falls including interaction term - Spline models. 4](#_Toc152938199)

[S4 Fig. Association between blood pressure and falls, by whether participants used antihyptertensives including interaction term, adjusted for year 5](#_Toc152938200)

[S5 Fig. Association between blood pressure and falls, by whether participants had a diagnosis of cardiovascular disease including interaction term, adjusted for year 6](#_Toc152938201)

[S6 Fig. Association between blood pressure and falls, by whether participants died or not 7](#_Toc152938202)

[S7 Fig. Association between blood pressure and falls, by whether participants were frail or not 8](#_Toc152938203)

[S8 Table. Mixed-effects logistic regression on the linear association between falls and blood pressure 8](#_Toc152938204)

[S9 Table. Mixed-effects logistic regression on the piecewise linear association between falls and blood pressure with a knot at 130 mmHg 8](#_Toc152938205)

| S1 Fig. Plot showing the association between blood pressure and falls - Spline models. |
| --- |
| **1 Spline.** |
| **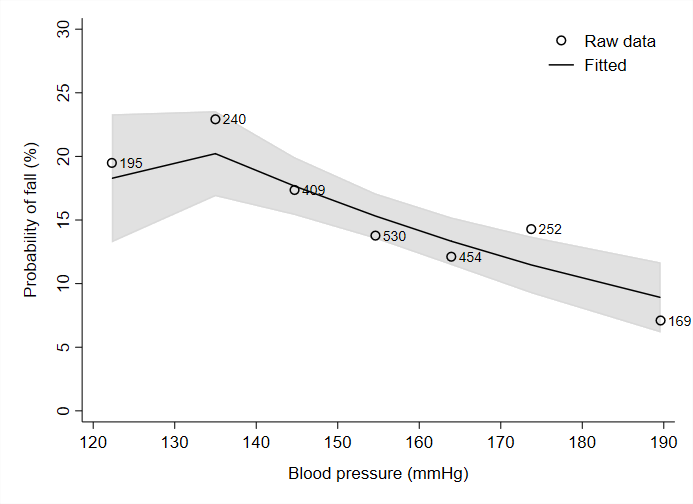** |
| **2 Splines.** |
| **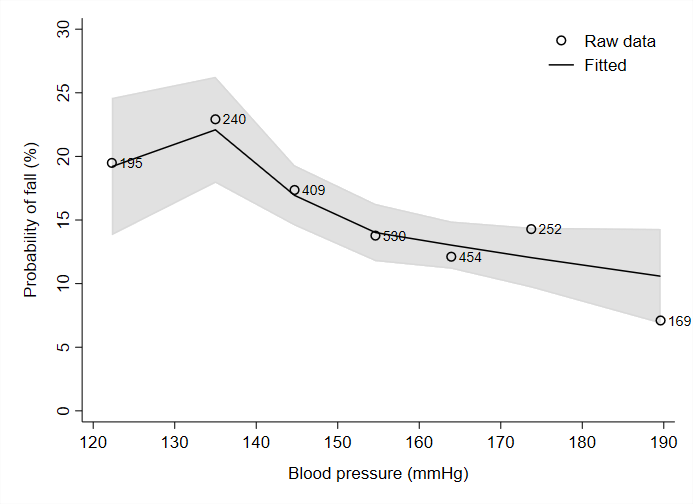** |
| The figures show the modelled probability to have at least one fall per year for different blood pressures (solid line) with 95% confidence band (shaded area). The points represent the raw data aggregated for blood pressures of <130, 130-140, 140-150, 150-160, 160-170 and >180 mmHg. |

| S2 Fig. Plot showing the association between blood pressure and the probability of falls, adjusted by year |
| --- |
| **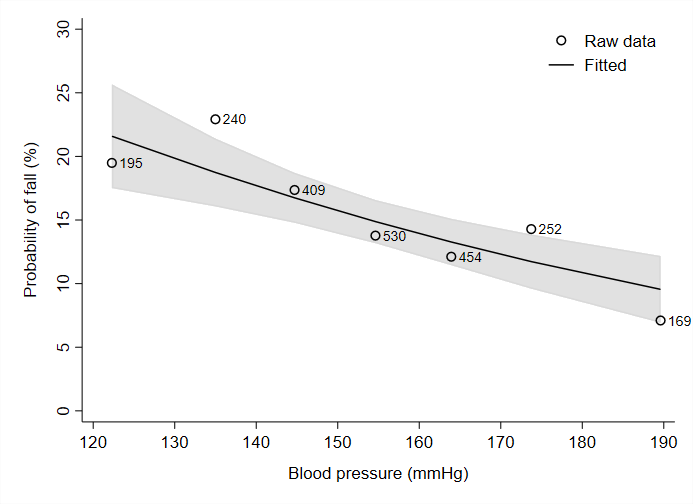** |
| The figure shows the modelled probability to have at least one fall per year for different blood pressures (solid line) with 95% confidence band (shaded area). The points represent the raw data aggregated for blood pressures of <130, 130-140, 140-150, 150-160, 160-170 and >180 mmHg. |

| S3 Fig. Plot showing the association between the use of antihypertensive medications and falls including interaction term - Spline models. |
| --- |
| **1 Spline.** |
| 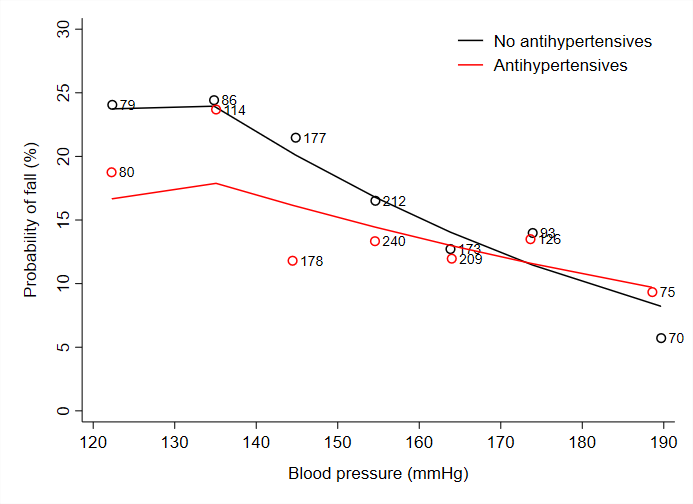 |
| **2 Splines.** |
| 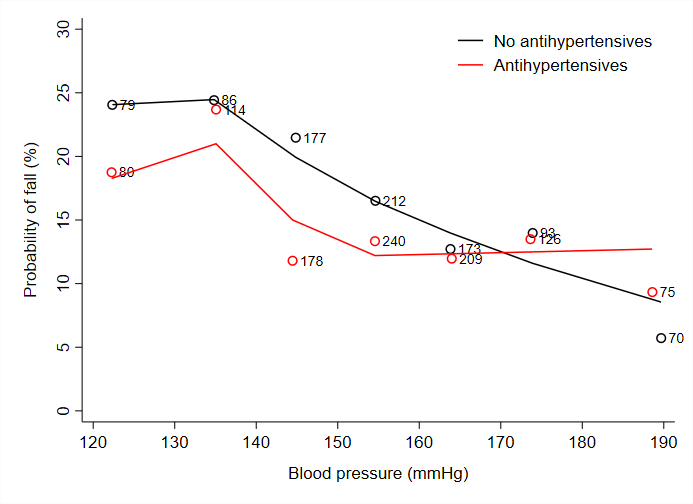 |
| The figures show the modelled probability to have at least one fall per year for different blood pressures (solid line) by antihypertensive use. The points represent the raw data aggregated for blood pressures of <130, 130-140, 140-150, 150-160, 160-170 and >180 mmHg. |

| S4 Fig. Association between blood pressure and falls, by whether participants used antihyptertensives including interaction term, adjusted for year |
| --- |
| **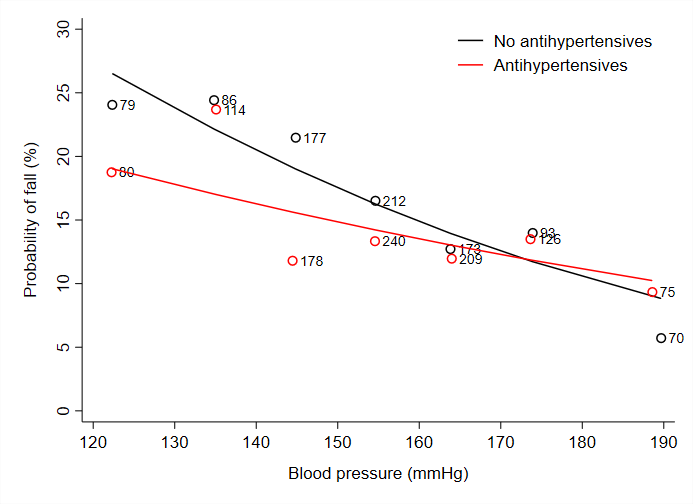** |
| The figure shows the modelled probability to have at least one fall per year for different blood pressures (solid line) by antihypertensive use. The points represent the raw data aggregated for blood pressures of <130, 130-140, 140-150, 150-160, 160-170 and >180 mmHg. |

| S5 Fig. Association between blood pressure and falls, by whether participants had a diagnosis of cardiovascular disease including interaction term, adjusted for year |
| --- |
| **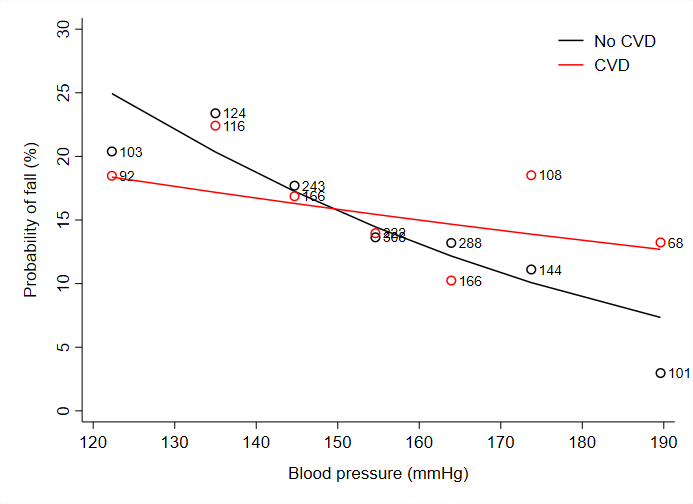** |
| The figure shows the modelled probability to have at least one fall per year for different blood pressures (solid line) by diagnosis of cardiovascular disease. The points represent the raw data aggregated for blood pressures of <130, 130-140, 140-150, 150-160, 160-170 and >180 mmHg. |
|  |

| S6 Fig. Association between blood pressure and falls, by whether participants died or not |
| --- |
|  |


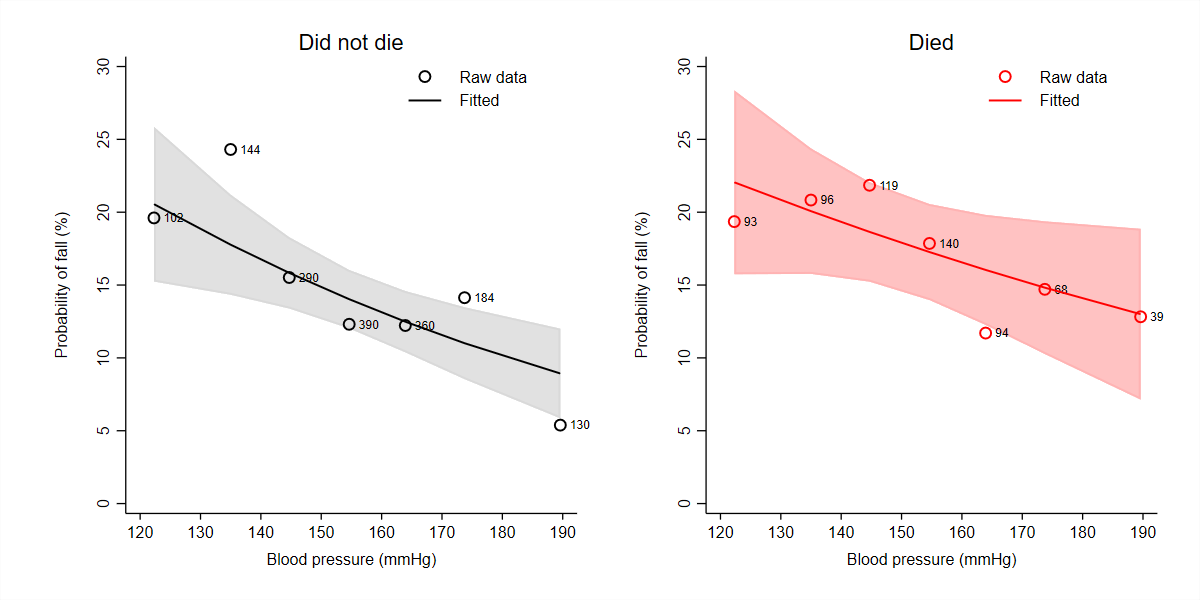


| S7 Fig. Association between blood pressure and falls, by whether participants were frail or not **(defined as lower than median hand grip strength or not able to perform a hand grip)** |
| --- |
|  |

**
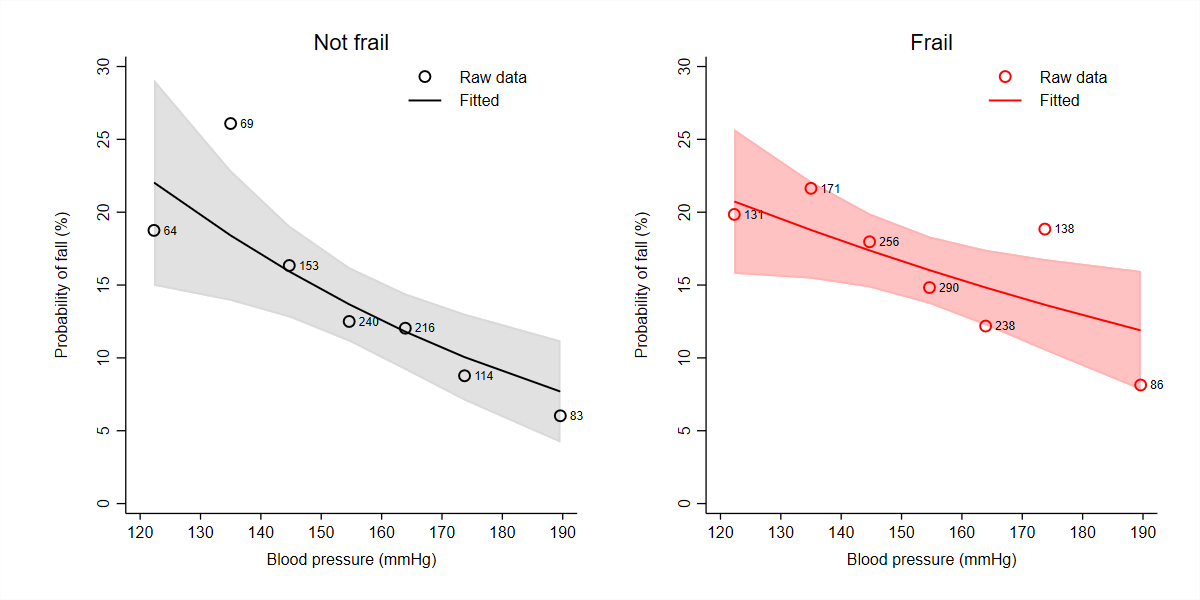
**

| S8 Table. Mixed-effects logistic regression on the linear association between falls and blood pressure (Number of observations=2249*, number of groups=544**) | | | |
| --- | --- | --- | --- |
| *Variables* | *Odds ratio (OR)* | *95% Confidence interval (CI)* | *p-Value* |
| Time-updated blood pressure | 0.86 | 0.80 – 0.93 | <0.001 |
| *over time, **groups=number of patients included in the analyses | | | |

| S9 Table. Mixed-effects logistic regression on the piecewise linear association between falls and blood pressure with a knot at 130 mmHg (Number of observations=2249*, number of groups=544**) | | | |
| --- | --- | --- | --- |
| *Variables* | *Odds ratio (OR)* | *95% Confidence interval (CI)* | *p-Value* |
| Time-updated blood pressure (<130 mmHg) | 1.35 | 0.81-2.26 | 0.25 |
| Time-updated blood pressure (>130 mmHg) | 0.83 | 0.76-0.91 | <0.001 |
| *over time, **groups=number of patients included in the analyses | | | |
